# Children’s visual acuity tests without professional supervision: a prospective repeated measures study

**DOI:** 10.1038/s41433-023-02597-7

**Published:** 2023-06-16

**Authors:** Daniel Osborne, Aimee Steele, Megan Evans, Helen Ellis, Roshni Pancholi, Tomos Harding, Jessica Dee, Rachel Leary, Jeremy Bradshaw, Elizabeth O’Flynn, Jay E. Self

**Affiliations:** 1 University Hospital Southampton NHS Foundation Trust, Department of Ophthalmology, Southampton, UK; 2 University of Southampton, Faculty of Medicine, Southampton, UK

**Keywords:** Physical examination, Paediatrics

## Abstract

**Background:**

Home visual acuity tests could ease pressure on ophthalmic services by facilitating remote review of patients. Home tests may have further utility in giving service users frequent updates of vision outcomes during therapy, identifying vision problems in an asymptomatic population, and engaging stakeholders in therapy.

**Methods:**

Children attending outpatient clinics had visual acuity measured 3 times at the same appointment: Once by a registered orthoptist per clinical protocols, once by an orthoptist using a tablet-based visual acuity test (iSight Test Pro, Kay Pictures), and once by an unsupervised parent/carer using the tablet-based test.

**Results:**

In total, 42 children were recruited to the study. The mean age was 5.6 years (range 3.3 to 9.3 years). Median and interquartile ranges (IQR) for clinical standard, orthoptic-led and parent/carer-led iSight Test Pro visual acuity measurements were 0.155 (0.18 IQR), 0.180 (0.26 IQR), and 0.300 (0.33 IQR) logMAR respectively. The iSight Test Pro in the hands of parents/carers was significantly different from the standard of care measurements (*P* = 0.008). In the hands of orthoptists. There was no significant difference between orthoptists using the iSight Test Pro and standard of care (*P* = 0.289), nor between orthoptist iSight Test Pro and parents/carer iSight Test Pro measurements (*P* = 0.108).

**Conclusion:**

This technique of unsupervised visual acuity measures for children is not comparable to clinical measures and is unlikely to be valuable to clinical decision making. Future work should focus on improving the accuracy of the test through better training, equipment/software or supervision/support.

## Introduction

Driven by the increasing number of outpatient appointments required and the wider availability of digital and communication technology, there is a shift in outpatient services towards innovative methods to communicate with patients and monitor their disease. The NHS long-term plan highlighted digital technology as a major facilitator of this shift, recommending that a third of outpatient appointments could be held virtually [1]. Ophthalmology is uniquely well suited to adopt this change because of the dramatic improvement in ocular imaging and the very high number of outpatient appointments. Mobile phone applications became commonplace in our society during the coronavirus pandemic with the NHS app enjoying 8.5 million downloads (2.6 million of which were in the month to May 2021 when the COVID pass functionality was introduced) since it was launched in January 2019 [2]. There are 45 apps that can be accessed with an NHS login with a variety of functions including: online pharmacy, e-referral, disease-specific information/support, interventions to improve health, and services to remotely monitor diseases [3].

Remote monitoring of traditionally clinical outcomes is already standard practice for measures such as blood pressure and glucose levels for people with hypertension and diabetes respectively. In ophthalmology, the USA Food and Drug Administration (FDA) have approved medical devices to monitor diabetic retinopathy and age-related macular degeneration [4, 5]. Almost without exception, all patients seen by eye professionals have their visual acuity tested. In a medical setting, the measure is used to identify disease, quantify severity, recommend visual impairment registration, and monitor natural history of disease and/or response to treatment/therapy.

Amblyopia is a neuro-developmental condition with treatment plans that are based on visual acuity measures alone. Up to 5% of children have amblyopia, it costs £1365 to treat a child and up to 50% of children have an unsuccessful outcome from gold standard therapy (occlusion therapy) [6, 7]. Amblyopia is associated with anisometropia, strabismus, or any condition or disease that insults vision during the critical period of visual development, which begins at birth and continues to between age 7 & 12 years [8]. Many diagnoses are made from vision screening of age 4–5 year-old schoolchildren [9]. In England, the National Screening Committee (NSC) recommend this age group achieve 0.20 logMAR (6/9 Snellen equivalent) visual acuity or better; those that do not should be “referred on for assessment of ocular motility and binocular function, cycloplegic refraction, and examination of optical media and retina/fundus” [10]. Some patients may present younger as their family seek referral for symptoms such as appearance of strabismus or concern for their child’s visual behaviour.

Home visual acuity tests for children may not be accurate enough to be relied upon when considering whether an amblyopia patient should be seen in clinic, or therapy should start or end [11]. Inaccuracies could be caused by change in test distance, peeking around occlusive glasses/eye patch, examiners offering children cues, or early termination of the test as the child loses interest. When operated by professionals, computerised visual acuity tests (apps) give measurements comparable to their traditional printed counterparts [12]. A systematic review identified 14 studies published before April 2020 that compared home-based to clinical standard visual acuity tests [13]. Three of the included studies recruited children, all of which found good agreeability between the home-based and clinical standard tests. The home-based tests were operated by a professional for two of the studies [14, 15], and by a trained school teacher for the third [16]. The review did not identify any studies in which parents/carers tested their children’s visual acuity with or without supervision from professionals.

Supervision of children and their parent/carer as they complete the visual acuity test may be virtual or in person, requiring time commitment from stretched clinicians. An unsupervised test has more possible uses to health services and patients. Several studies published after 2020, using a variety of apps, have compared unsupervised parent/carer-led visual acuity tests with gold-standard visual acuity tests [17–21], finding good to moderate agreement. The primary aim of our study was to collect data about the accuracy of unsupervised parent/carer-led, visual acuity tests of their children using widely available, clinically validated tablet-based software (apps) without training nor software/equipment purposefully designed for parent/carer home use. A secondary outcome was to collect quantitative data about families’ access to the required equipment and technology. Additionally, in a questionnaire, we asked families if they found the tests easy and whether the outputs of the data would be helpful to them during their child’s care.

## Materials and methods

### Study design and participants

Children between age 3 and 10 years were identified from orthoptic and paediatric ophthalmology outpatient clinics by a member of the research team (DO, ME, or AS). Recruitment ran from July 2020 to November 2021 and sampling was dependant on availability of research staff, equipment, and outpatient clinic capacity. Children were excluded from the visual acuity data collection protocol if they were unable to complete a clinical subjective visual acuity test, if their parent or carer was under age 17 years (a regulatory requirement of the Kay Pictures iSight Test Pro software), or they or their parent/carer was not willing to give informed assent/consent.

Upon consent, children were assigned a sequential, unique study identifier (USI) number. Each participant completed three visual acuity tests on the same day in the clinic office:

- A standard of care, clinical visual acuity test. A registered orthoptist tested visual acuity as per clinical guidelines and experience. Orthoptists selected an age and level of understanding appropriate test from: Single of Linear Crowded Kay Pictures (Tring, UK) book, Keeler (Windsor, UK) logMAR book or Bailey-Lovie letters on a Thomson (Welham Green, UK) Test Chart.
- An orthoptist-led, tablet computer-based (Apple iPad, Apple, California, USA; iSight Test Pro, Kay Pictures, Tring, UK), visual acuity test. The same orthoptist that completed the standard of care test measured the participant’s visual acuity using the iSight Test Pro and an Apple iPad. As they completed the test (approximately 10 min), they showed the parent or carer how to:
  load the application
  select the appropriate visual acuity test
  Measure the correct distance to perform the test
  Effectively occlude one eye (using either occlusive glasses or Durapore over one lens of spectacles) for uniocular testing
  Complete the test and record the result
- A trained parent/carer-led, tablet computer-based visual acuity test. Following observation of use of the iPad and iSight Test Pro, and a short training session, the parent was asked to measure their child’s visual acuity. They were left alone in a clinical room for up to 15 min. The room had a 3-metre distance from the patient marked on the floor.

Children with odd USI numbers completed the tests in the order they appear above (*abc*), whereas those with even USI numbers used a *bac* order. This aimed to reduce order effects as children tire through the testing procedures but give parents/carers opportunity to see the iSight Test Pro app in use prior to using it themselves. Following completion, the participant returned to clinical care, the parent or carer completed a short questionnaire (Table 1), and visual acuity results were collated for analysis.

**Table 1:** Demographics, characteristics of participants and questionnaire responses.

Sex	25 males / 17 females	
Age at testing mean ± SD, (range)	5 years 7 months ± 15 months, (41–113 months)	
Ocular diagnosis N (%)	Amblyopia	13 (31)
	Hypermetropia	13 (31)
	No ocular disease	4 (9.5)
	Astigmatism	3 (7.1)
	Nystagmus	3 (7.1)
	Myopia	2 (4.8)
	Intermittent distance exotropia	2 (4.8)
	Infantile cataract	1 (2.4)
	Vernal keratoconjunctivitis	1 (2.4)
Do you think you could do the test at home with your child? N (%)	Yes	40 (97.6)
	No	1 (2.4)
Which of the following devices do you have daily access to at home? (Select all that apply) N (%)	Apple iPad	18 (23.4)
	Android tablet	20 (26.4)
	Android smartphone	14 (18.2)
	Apple iPhone	23 (29.9)
	Windows smartphone	2 (2.6)
How easy did you find doing the test with your child? N (%)	Very difficult	0 (0.0)
	Difficult	1 (2.4)
	Easy	21 (51.2)
	Very easy	19 (46.3)
How helpful would you find the information from a home visual acuity assessment through the course of your child’s treatment? N (%)	Very unhelpful	0 (0.0)
	Not helpful	2 (4.9)
	Helpful	24 (58.5)
	Very helpful	15 (36.5)

### Statistical analysis

Visual acuity data from each participant’s right eye only were used in statistical analysis. The right eye was tested first in the standard of care tests, reducing the effects of test fatigue. Comparisons between standard of care, orthoptist-led iSight Test Pro, and parent/carer iSight Test Pro visual acuity data were made using Kruskall-Wallis tests with *P* values calculated using a post hoc Dunn Test. Limits of Agreement (LOA) were calculated with quantile regression and bootstrapping used to estimate confidence intervals (i.e., systematic bias). We used linear regression to assess the effect of worsening visual acuity on test accuracy (i.e., proportional bias).

## Results

### Characteristics of participants

In total, 42 children were recruited to the study (Table 1). The mean age was 5 years 7 months (SD 15 months, range 3 years 4 months to 9 years 4 months). 25 were male, 17 females, 13 were under frequent outpatient follow-up for amblyopia therapy (occlusion or atropine penalisation therapy). Visual acuity data were collected for both eyes for all participants in accordance with the testing protocol.

### Differences between visual acuity measurements (systematic bias)

**Fig. 1:**
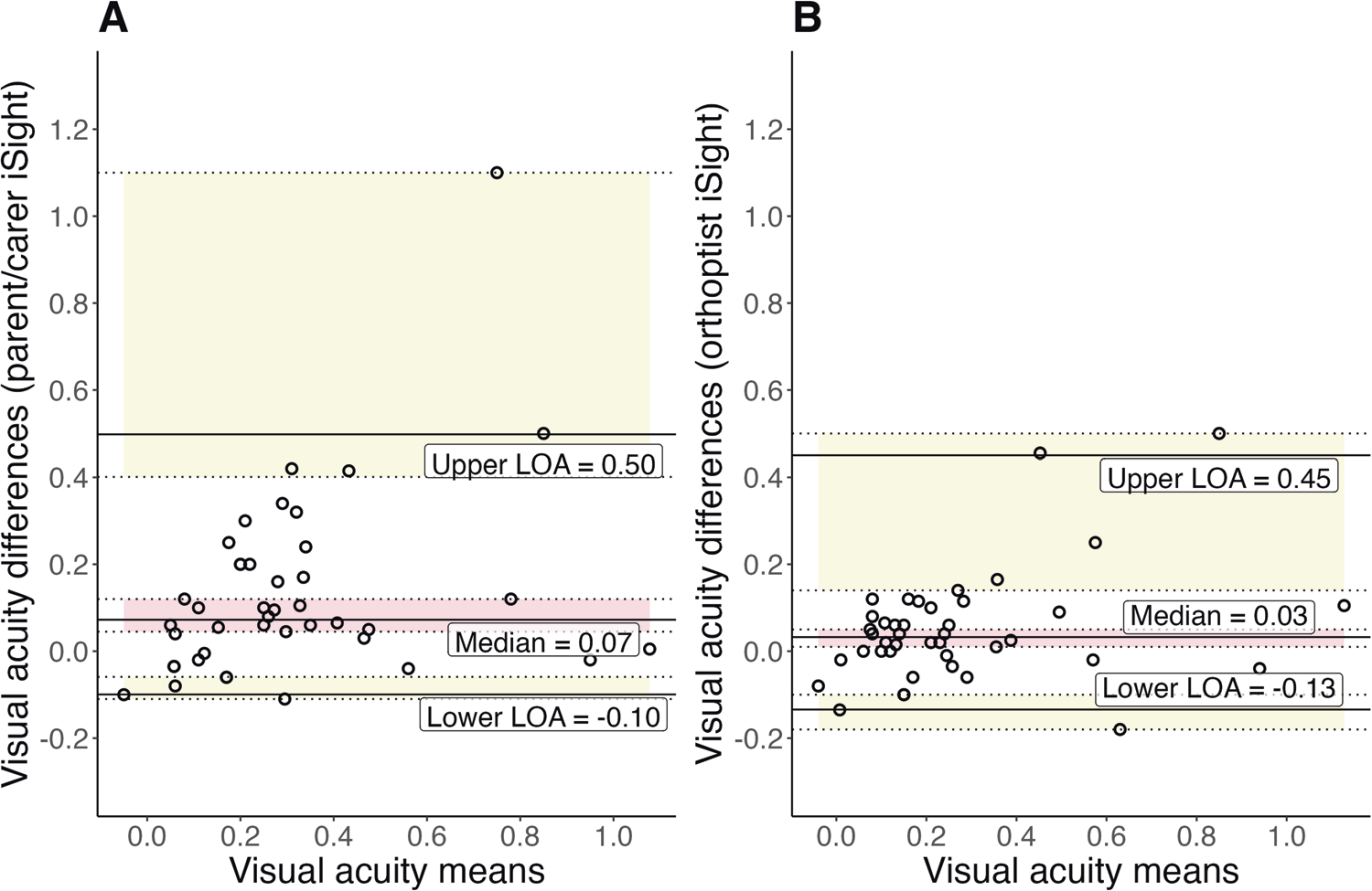
Systematic bias of parent/carer-measured and orthoptist-measured iSight Test Pro visual acuity. Modified Bland-Altman plots for parent/carer measured iSight Test Pro (**A**) and orthoptist measured iSight (**B**) visual acuity against standard of care measurements. Description: X-axis = mean of iSight Test Pro and standard of care measurements. Y-axis = iSight Test Pro minus standard of care measurements. Systematic bias = median difference between iSight Test Pro and standard of care tests. Limit of Agreement (LOA) = 2.5% and 97.5% quantiles denoted by solid black upper and lower lines. Confidence intervals (CI) = 90% CI (bootstrapping) for LOA and 95% CI for median systematic bias denoted by dotted lines and shading.

The median values and interquartile ranges for the clinical standard, and orthoptist and parent/carer iSight Test Pro were 0.155 (IQR = 0.095-0.275), 0.180 (IQR = 0.100–0.360), and 0.300 (IQR = 0.135–0.465) logMAR respectively (Table 2). The Kruskall-Wallis test showed that the three tests significantly differed (P = 0.03, ***Χ***^*2*^ = *7.21*). Post hoc Dunn Test showed the iSight Test Pro in the hands of parents/carers gave significantly poorer acuities than the standard of care measurements (*P* = 0.008), but in the hands of orthoptists, there was no significant difference between the iSight Test Pro app and standard of care (*P* = 0.289), nor was there significant difference between parents/carers and orthoptists using iSight Test Pro (*P* = 0.108). Modified Bland-Altman plots show greater variation of the differences between parent/carer iSight Test Pro and standard care than between orthoptist iSight Test Pro and standard care (Fig. 1). The median bias of the orthoptist iSight Test Pro against standard care tests was 0.07 logMAR (95% confidence interval (CI): 0.04 to 0.12). The lower limits of agreement (LOA) was −0.10 (90%CI: −0.11 to −0.06) and upper LOA 0.50 (90% CI: 0.40–1.10). The orthoptist iSight Test Pro against standard clinical care median bias was 0.03, lower LOA was −0.13 (90%CI: −0.18 to −0.10) and upper LOA was 0.45 (90%CI: 0.14–0.50).

**Table 2:** Orthoptists and parent/carer’s iSight Test Pro measurements compared to standard clinical care measurements.

Difference between iSight test pro and clinical tests	Orthoptist iSight test pro measure N (%)	Parent/carer iSight test pro measure N (%)
Within 1 line	53 (63.1)	39 (46.4)
1–2 lines	23 (27.4)	21 (25.0)
2–3 lines	6 (7.1)	12 (14.3)
3–4 lines	0 (0.0)	4 (4.8)
4–5 lines	1 (1.2)	5 (6.0)
5–6 lines	1 (1.2)	1 (1.2)
Greater than 6 lines	0 (0.0)	2 (2.4)
Median difference to clinical standard (25th–75th percentile), logMAR	0.060 (0.020–0.120)	0.100 (0.045–0.200)
Range of differences to clinical standard, logMAR	0–0.500	0–1.280

*N* = 84 eyes, 42 participants.

### Correlation between level of visual acuity and accuracy of iSight Pro Tests (proportional bias)

**Fig. 2:**
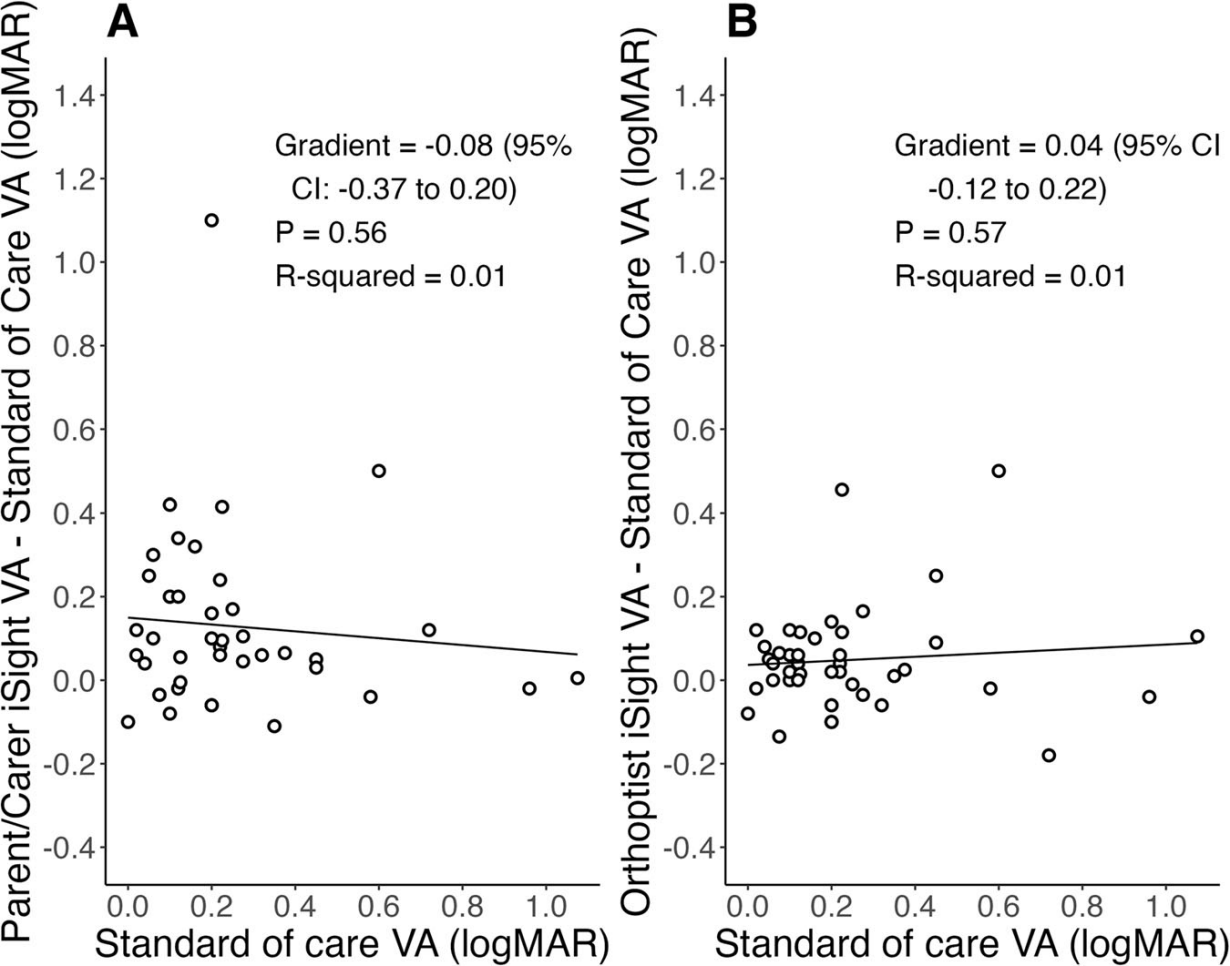
Proportional bias of parent/carer-measured and orthoptist-measured iSight Test Pro visual acuity. Linear regression of (**A**) parent/carer iSight Test Pro and (**B**) orthoptist iSight Test Pro against standard of care visual acuity. Description: The 95% confidence intervals (CI) of the slope gradient span 0; the null hypothesis that level of standard of care visual acuity does not affect iSight Test Pro measure is accepted.

There was no correlation between worsening (increasing) standard of care visual acuity and difference between standard of care test and parent/carer iSight Test Pro (*R*^*2*^ = 0.01, *P* = 0.56) nor orthoptist iSight Test Pro (*R*^*2*^ = 0.01, *P* = 0.57) measures (Fig. 2).

### Outliers

Outliers in our data could skew the results towards the conclusion that the iSight Test Pro underestimates visual acuity. We defined outliers as a measurement greater than 0.50 logMAR units from the standard of care measurements and describe data for each outlier below:

- Participant 24 is a 4-year-old boy under follow-up for bilateral hypermetropia. They completed the full data collection procedure with no noted protocol deviations, including orthoptist iSight Test Pro, followed by standard of care and finally parent/carer iSight Test Pro measurements. The parent/carer iSight Test Pro (1.30 logMAR) was substantially different from the orthoptist iSight Test Pro (0.10 logMAR) and standard of care (0.20 logMAR) measurements. In completing the questionnaire, the parent indicated the test had been “difficult” to complete.
- Participant 25 is a 9-year-old boy under follow up for unilateral mixed strabismic-anisometropic amblyopia and has known reduced right eye visual acuity related to their condition. Their standard of care visual acuity was 0.60 logMAR and both the parent/carer and orthoptist iSight Test Pro measured the visual acuity as 1.10 logMAR.

## Discussion

In this study, we compare the accuracy of parent/carer-led, tablet-based VA tests to clinical standard, and orthoptic-led tablet-based tests. Parents were left unsupervised in a private room to simulate a home environment. They were shown how to use the iSight Test Pro, maintain the correct testing distance using a permanent mark on the floor, but were not given any live feedback on their testing technique. This approach resulted in measurements that could not be compared to clinical measures; parent/carer measured VA differed significantly from standard of care measurements. There were two outliers in our data (parent measurements ⩾5 lines different from standard of care) that could have occurred for a variety of reasons. Firstly, the parent/carer test was always collected after the participant had had their visual acuity measured twice by an orthoptist, risking test fatigue or loss of interest in the test. There was no evidence in our data that younger children or those with worse visual acuity were less likely to have an accurate parent/carer test. Parent/ carer iSight Test Pro measures were comparable to measures collected by professionals using the same equipment and software, suggesting that these factors, as well as testing technique, play a role in this difference.

There have been a variety of teams working to develop new equipment and methods of testing children’s visual acuity in a home setting. The *Amblyopia Tracker App* (Kay Pictures) and *DigiVis* are apps that attempt to control the variables of a typical visual acuity test by only allowing users to alter distance and not optotype size, and by measuring distance with a second device respectively [21, 22]. Both have good agreeability with clinical standard tests but further work on their utility and implementation is required. The *Peekaboo Vision* [23] and *OKKO health* [24] are apps that measure near visual acuity and gamify the test. In Peekaboo Vision, children are presented with a grey screen with a grating stimulus in one corner. When the child touches the stimulus, they are rewarded as the stimulus transforms through animation into a smiling face. The test is made progressively harder through finer gratings until visual acuity threshold. Children appear to enjoy this method and it may have utility in visual acuity tests for children with Special Educational Needs. It is currently unknown which methods may encourage families to complete home visual acuity testing [25].

Families appear reluctant to use visual acuity testing apps at home. Painter et al. (2021) [26] contacted 103 parents or carers by telephone, inviting them to use a VA test at home with their children after their outpatient appointment. 96 families confirmed they would take part, but only 15 families (14.6%) completed the test and returned the results. Common reasons for not completing were lack of time or did not understand / receive the written instructions by email. Thirunavukarasu et al. (2021) [17] had similar problems with their DigiVis app, inviting 511 patients to take part in a research study of home vision tests, with 120 responding and participating (23%). DigiVis requires two devices, which may exclude some families that do not have access to equipment.

Access to equipment could be an important barrier to implantation of home vision testing. Children from the lowest socio-economic class are 1.82x more likely to have amblyopia [6] and may be less likely to adhere to current therapies [27, 28]. When planning service provision, policymakers should target this group, which are the least likely to have access to expensive equipment. All respondents to our parent/carer questionnaire had access to at least one device capable of running a VA test app at home, suggesting it unlikely that access to equipment is a significant barrier to use of the tests. Furthermore, parents and carers appeared to appreciate the usefulness of home-collected data and did not feel the test process would be challenging to do at home. All our participants received one to one demonstration of the app immediately prior to using it themselves. Future studies should look at offering parents/carers a demonstration in the clinic with the test completed later at home compared to written information delivered through email. Further qualitative work to evaluate the process of home VA tests may identify areas for improvement in the implementation of these tests.

Clinicians and services also have reservations about widespread use of home vision tests. In June 2020, The Royal College of Ophthalmologists and British and Irish Orthoptic Society (BIOS) published a joint statement warning their members: “The reliability of apps when used by a parent or guardian in the home setting to test visual acuity in children is not yet proven” [11]. A lack of reliability could lead to patients receiving appointments unnecessarily or not being seen when they ought to have been. Our data does little to absolve this notion with differences between clinical standard and home iSight Test Pro measurements likely caused by a combination of limited parent/carer training, outliers, and differences in equipment between the home and clinic tests.

Our data suggests that while some parents/carers can test their children’s visual acuity using a clinic-like app, some may struggle. Erroneous measures may not always be possible to detect and as such we do not think these tests should be used without supervision from a professional, and with limited training only. Newer generations of tests are emerging that can control variables inherent to traditional visual acuity tests and/or gamify the process. A third approach is to modify the tests to include remote/virtual supervision of parents/carers and their children by clinicians/professionals during the home VA test. It remains to be seen which approach becomes favoured for implementation into clinical practice. We highlight the need for engaging tests to increase the rate of uptake particularly among families from lower socio-economic backgrounds. Additionally, researchers and developers should be mindful of what equipment is required for patients to access new visual acuity tests.

### Limitations

Our study has limitations that could affect the results and conclusions. The clinic environment in which the home simulated (parent/carer-led) visual acuity tests were completed is set up to accurately test children’s eyes and VA. The room provides even, bright lighting, markers on the floor to specify test distance, and isolation from distractions that may be present in the home. The parent/carer-led test was always completed last after participants had had their visual acuity measured twice already. It is likely that this age group will have lost interest by this point, skewing the parent/carer test results.

## Summary

### What was known before

- Visual acuity tests for children at home could improve problems faced by outpatient services and improve patient care. Current available tablet or phone-based tests, operated or supervised by an eye professional, are comparable to gold-standard clinical tests.

### What this study adds

- We show that unsupervised tests led by parents or carers may not be as accurate as gold-standard clinical tests. Innovative improvements to the home tests to make them more engaging and/or control for variables such as test distance are required.

## Data Availability

The data that supports the findings of this work are available from the corresponding author upon reasonable request.
